# Supplementary material for: Risk factors associated with rhinitis, allergic conjunctivitis and eczema among schoolchildren in Uganda
Source: Clin Exp Allergy. Author manuscript; Available in PMC 2021 Dec 14. (PMC7612100; doi:10.1111/cea.13769)
Supplement: Table S1-S4 [file EMS140521-supplement-Table_S1_S4.docx]

**Supplementary Table 1: Risk factors for rhinitis among schoolchildren in Uganda (N=1,692)**

| Characteristics | Rhinitis ever | | Adj. OR^‡^ (95% CI) | P-value |
| --- | --- | --- | --- | --- |
|  | **Yes** (N=870) | **No** (N=822) |  |  |
| Age, years Mean (SD) | 11.51 (3.12) | 10.65 (3.08) | 1.08 (1.04-1.12) | <0.0001 |
| Girls (942) | 511 (58.7) | 431 (52.4) | 1.39 (1.12-1.74) | 0.003 |
| Father’s education [m=21] |  |  |  |  |
| None/Primary (437) | 198 (22.9) | 239 (29.6) | 1 |  |
| Secondary (595) | 307 (35.6) | 288 (35.6) | 1.14 (0.87-1.50) |  |
| Tertiary (639) | 358 (41.5) | 281 (34.8) | 1.37 (1.03-1.80)^a^ | 0.08 |
| Father’s history of allergic disease [m=149] | | | | |
| Yes (363) | 242 (30.1) | 121 (16.4) | 2.08 (1.57-2.75) | <0.0001 |
| Mother’s history of allergic disease [m=126] | | | | |
| Yes (592) | 384 (46.9) | 208 (27.8) | 2.29 (1.81-2.91) | <0.0001 |
| Area of residence at the time of the child’s birth [m=1] | | | | |
| Rural (354) | 159 (18.3) | 195 (23.7) | 1 |  |
| Town (1,188) | 612 (70.3) | 576 (70.2) | 1.22 (0.93-1.60) |  |
| City (149) | 99 (11.4) | 50 (6.1) | 1.97 (1.26-3.10)^b^ | 0.01 |
| Exposure to animals in first five years of life [m=57] | | | | |
| Yes (615) | 331 (39.2) | 284 (36.0) | 1.23 (0.98-1.55) | 0.07 |
| Frequency of ‘trucks passing on street near child’s home’ at enrolment [m=1] | | | | |
| Rarely (944) | 442 (50.9) | 501 (61.0) | 1 |  |
| Frequently (624) | 346 (39.8) | 278 (33.9) | 1.42 (1.13-1.79) |  |
| Almost all the time (123) | 81 (9.3) | 42 (5.1) | 1.90 (1.19-3.03)^c^ | 0.001 |
| Use of de-worming medication in last 12 months [m=1] | | | | |
| None (578) | 276 (31.8) | 302 (36.8) | 1 |  |
| Once (732) | 345 (39.7) | 387 (47.1) | 0.85 (0.67-1.09) |  |
| >Twice (380) | 248 (28.5) | 132 (16.1) | 1.80 (1.32-2.45)^d^ | <0.0001 |
| Any helminth infection at enrolment [m=130] | | | | |
| Yes (212) | 90 (11.2) | 122 (16.0) | 0.70 (0.50-0.97) | 0.03 |
| Tuberculin skin test induration at enrolment [N=960] | | | | |
| Positive (>10mm) (143) | 66 (13.4) | 77 (16.4) | 0.66 (0.44-1.01) | 0.05 |
| Skin Prick Test response to any of 7 allergens at enrolment [m=36] | | | | |
| Positive (>3mm) (657) | 392 (45.8) | 265 (33.1) | 1.56 (1.24-1.96) | <0.0001 |
| Fractional exhaled nitric oxide levels at enrolment [m=80] | | | | |
| Elevated (>35ppb) (381) | 254 (30.2) | 127 (16.4) | 2.06 (1.57-2.70) | <0.0001 |
| Allergen-specific IgE to any of 3 allergens at enrolment (N=397)^§^ | | | | |
| Atopic (>0.35 kU_A_/L) (251) | 142 (66.4) | 109 (59.6) | 1.33 (0.81-2.18) | 0.26 |
| Total IgE (N=398) Median (95%CI) kU/L | 414.8 (281.1-537.5) | 304.3 (213.6-359.3) | 1.00002 (0.99983-1.00020) | 0.87 |

N=number; Adj. OR=adjusted odds ratio; CI=confidence interval; SD=standard deviation; m=missing; Columns 2 and 3 represent number (%), unless stated otherwise. ^‡^Adjusted for child’s age, sex, area of residence at birth and father’s education. Test for trend p-value: ^a^=0.03; ^b^=0.006; ^c^<0.0001; ^d^=0.002. ^§^ ImmunoCAP^®^, cut-off >0.35 kU_A_/L.

**Supplementary Table 2: Risk factors for currently having any of rhinitis, conjunctivitis, eczema and asthma among Ugandan schoolchildren (N=1,693)**

| Characteristics | Allergy-related disease in last 12-months | | Adj. OR^‡^ (95% CI) | P-value |
| --- | --- | --- | --- | --- |
|  | **Yes** (N=734) | **No** (N=959) |  |  |
| Age, years Mean (SD) | 11.34 | 10.91 | 1.05 (1.01-1.09) | 0.01 |
| Girls (942) | 388 (52.9) | 554 (57.8) | 0.83 (0.66-1.03) | 0.09 |
| Father’s history of allergic disease [m=150] | | | | |
| Yes (363) | 217 (32.2) | 146 (16.8) | 2.06 (1.57-2.70) | <0.0001 |
| Mother’s history of allergic disease [m=128] | | | | |
| Yes (592) | 312 (45.3) | 280 (31.9) | 1.66 (1.31-2.11) | <0.0001 |
| Area of residence at the time of the child’s birth [m=1] | | | | |
| Rural (355) | 117 (16.0) | 238 (24.8) | 1 |  |
| Town (1,188) | 531 (72.4) | 657 (68.5) | 1.32 (0.99-1.77) |  |
| City (149) | 85 (11.6) | 64 (6.7) | 1.91 (1.22-2.99)^a^ | 0.01 |
| Child’s area of residence in first five years of life [m=1] | | | | |
| Rural (339) | 110 (15.0) | 229 (23.9) | 1 |  |
| Town (1,267) | 570 (77.8) | 697 (72.7) | 1.41 (1.04-1.89) |  |
| City (86) | 53 (7.2) | 33 (3.4) | 1.78 (1.03-3.06)^b^ | 0.04 |
| Exposure to animals in first five years of life [m=60] | | | | |
| Yes (615) | 276 (38.8) | 339 (36.8) | 1.45 (1.14-1.84) | 0.002 |
| Frequency of ‘trucks passing on street near child’s home’ at enrolment [m=1] | | | | |
| Rarely (945) | 370 (50.5) | 575 (60.0) | 1 |  |
| Frequently (624) | 287 (39.1) | 337 (35.1) | 1.18 (0.93-1.49) |  |
| Almost all the time (123) | 76 (10.4) | 47 (4.9) | 2.15 (1.38-3.35)^c^ | 0.003 |
| Use of de-worming medication in last 12 months [m=3] | | | | |
| None (578) | 210 (28.7) | 368 (38.4) | 1 |  |
| Once (732) | 304 (41.6) | 428 (44.6) | 1.16 (0.89-1.50) |  |
| >Twice (380) | 217 (29.7) | 163 (17.0) | 1.87 (1.38-2.53)^d^ | 0.0002 |
| Any helminth infection at enrolment [m=130] | | | | |
| Yes (212) | 78 (11.5) | 134 (15.1) | 0.87 (0.61-1.24) | 0.45 |
| Skin Prick Test response to any of 7 allergens at enrolment [m=36] | | | | |
| Positive (>3mm) (657) | 364 (50.6) | 293 (31.3) | 1.70 (1.35-2.14) | <0.0001 |
| Fractional exhaled nitric oxide levels at enrolment [m=80] | | | | |
| Elevated (>35ppb) (381) | 238 (33.2) | 143 (16.0) | 1.99 (1.53-2.59) | <0.0001 |
| Allergen-specific IgE at enrolment (N=398)^§^ | | | | |
| Atopic (>0.35 kU_A_/L) (252) | 159 (71.3) | 93 (53.1) | 1.85 (1.14-3.01) | 0.01 |
| Total IgE (N=398) Median (95%CI) kU/L | 432.4 (306.5-595.7) | 274.9 (207.4-339.7) | 1.0001 (0.9999-1.0003) | 0.23 |

N=number; Adj. OR=adjusted odds ratio; CI=confidence interval; SD=standard deviation; m=missing; Columns 2 and 3 represent number (%), unless stated otherwise. ^‡^Adjusted for child’s age, sex, area of residence at birth and father’s education. Test for trend p-valve: ^a^=0.004; ^b^=0.01; ^c^=0.002; ^d^<0.0001. ^§^ImmunoCAP^®^ cut-off >0.35 kU_A_/L.

**Supplementary Table 3: Risk factors for having ever had any of rhinitis, conjunctivitis and eczema among Ugandan schoolchildren without asthma (N=1,132)**

| Characteristics | Allergy-related disease ever | | Adj. OR^‡^ (95% CI) | P-value |
| --- | --- | --- | --- | --- |
|  | **Yes** (N=634) | **No** (N=498) |  |  |
| Age, years Mean (SD) | 11.19 | 10.64 | 1.05 (1.01-1.09) | 0.02 |
| Girls (646) | 384 (60.6) | 262 (52.6) | 1.37 (1.07-1.75) | 0.01 |
| Father’s education [m=17] |  |  |  |  |
| None/Primary (338) | 183 (29.2) | 155 (31.8) | 1 |  |
| Secondary (401) | 231 (36.8) | 170 (34.8) | 1.13 (0.84-1.51) |  |
| Tertiary (376) | 213 (34.0) | 163 (33.4) | 1.16 (0.32-1.00)^a^ | 0.61 |
| Father’s history of allergic disease [m=113] | | | | |
| Yes (188) | 138 (24.0) | 50 (11.2) | 2.44 (1.71-3.48) | <0.0001 |
| Mother’s history of allergic disease [m=95] | | | | |
| Yes (346) | 237 (40.1) | 109 (24.4) | 2.10 (1.59-2.77) | <0.0001 |
| Area of residence at the time of the child’s birth | | | | |
| Rural (280) | 159 (25.1) | 121 (24.3) | 1 |  |
| Town (772) | 419 (66.1) | 353 (70.9) | 0.96 (0.72-1.27) |  |
| City (80) | 56 (8.8) | 24 (4.8) | 1.88 (1.09-3.25)^b^ | 0.02 |
| Exposure to animals in first five years of life [m=46] | | | | |
| Yes (426) | 265 (43.4) | 161 (33.8) | 1.49 (1.15-1.93) | 0.002 |
| Frequency of ‘trucks passing on street near child’s home’ at enrolment | | | | |
| Rarely (676) | 357 (56.3) | 319 (64.1) | 1 |  |
| Frequently (392) | 232 (36.6) | 160 (32.1) | 1.30 (1.01-1.69) |  |
| Almost all the time (64) | 45 (7.1) | 19 (3.8) | 1.92 (1.08-3.42)^c^ | 0.02 |
| Use of de-worming medication in last 12 months [m=1] | | | | |
| None (427) | 233 (36.8) | 194 (39.0) | 1 |  |
| Once (500) | 259 (40.9) | 241 (48.4) | 0.90 (0.69-1.17) |  |
| >Twice (204) | 141 (22.3) | 63 (12.6) | 1.91 (1.33-2.74)^d^ | 0.0001 |
| Any helminth infection at enrolment [m=87] | | | | |
| Yes (157) | 78 (13.2) | 79 (17.3) | 0.76 (0.53-1.07) | 0.12 |
| Skin Prick Test response to any of 7 allergens at enrolment [m=26] | | | | |
| Positive (>3mm) (354) | 226 (36.4) | 128 (26.3) | 1.66 (1.27-2.17) | <0.0001 |
| Fractional exhaled nitric oxide levels at enrolment [m=66] | | | | |
| Elevated (>35ppb) (181) | 121 (19.8) | 60 (13.2) | 1.61 (1.14-2.27) | 0.007 |
| Allergen-specific IgE at enrolment (N=199)^§^ | | | | |
| Atopic (>0.35 kU_A_/L) (109) | 58 (56.3) | 49 (51.0) | 1.20 (0.68-2.14) | 0.53 |
| Total IgE (N=398) Median (95%CI) kU/L | 234.8 (178.3-328.5) | 310.0 (187.6-381.5) | 0.9997 (0.9994-1.0000) | 0.06 |

N=number; Adj. OR=adjusted odds ratio; CI=confidence interval; SD=standard deviation; m=missing; Columns 2 and 3 represent number (%). ^‡^Adjusted for child’s age, sex, area of residence at birth and father’s education. Test for trend p-valve: ^a^=0.76; ^b^=0.17; ^c^=0.005; ^d^=0.006. ^§^ImmunoCAP^®^ cut-off >0.35 kU_A_/L.

**Supplementary Table 4: Risk factors for urticarial rash among schoolchildren in Uganda (N=1,691)**

| Characteristics | Urticarial rash ever | | | Adj. OR^‡^ (95% CI) | P-value |
| --- | --- | --- | --- | --- | --- |
|  | **Yes** (N=572) | **No** (N=1,119) | |  |  |
| Age, years Mean (SD) | 12.01 (3.04) | | 10.64 (3.07) | 1.18 (1.13-1.23) | <0.0001 |
| Girls (942) | 339 (59.3) | | 603 (53.9) | 1.16 (0.91-1.47) | 0.22 |
| Fathers’ highest education attained [m=20] | | | | | |
| None/Primary (437) | 168 (29.5) | | 269 (24.4) | 1 |  |
| Secondary (595) | 195 (34.3) | | 400 (36.3) | 0.71 (0.53-0.94) |  |
| Tertiary (639) | 206 (36.2) | | 433 (39.3) | 0.79 (0.59-1.05)^a^ | 0.06 |
| Father’s history of allergic disease [m=148] | | | | | |
| Yes (363) | 147 (27.8) | | 216 (21.3) | 1.68 (1.25-2.25) | 0.001 |
| Mother’s history of allergic disease [m=128] | | | | | |
| Yes (592) | 235 (43.8) | | 357 (34.7) | 1.58 (1.23-2.02) | <0.0001 |
| Area of residence at the time of the child’s birth | | | | | |
| Rural (354) | 126 (22.0) | | 228 (20.4) | 1 |  |
| Town (1,188) | 395 (69.1) | | 793 (70.9) | 1.15 (0.87-1.53) |  |
| City (149) | 51 (8.9) | | 98 (8.7) | 1.08 (0.67-1.74)^b^ | 0.60 |
| Exposure to animals in first five years of life [m=58] | | | | | |
| Yes (615) | 237 (42.9) | | 378 (35.0) | 1.29 (1.01-1.66) | 0.04 |
| Frequency of ‘trucks passing on street near child’s home’ at enrolment | | | | | |
| Rarely (944) | 292 (51.1) | | 652 (58.3) | 1 |  |
| Frequently (624) | 218 (38.1) | | 406 (36.3) | 1.19 (0.93-1.52) |  |
| Almost all the time (123) | 62 (10.8) | | 61 (5.4) | 2.19 (1.37-3.48)^c^ | 0.003 |
| Use of de-worming medication in last 12months [m=1] | | | | | |
| None (578) | 181 (31.7) | | 397 (35.5) | 1 |  |
| Once (732) | 237 (41.5) | | 495 (44.2) | 0.96 (0.74-1.25) |  |
| >Twice (380) | 153 (26.8) | | 227 (20.3) | 1.40 (1.02-1.92)^d^ | 0.04 |
| Any helminth infection at enrolment [m=129] | | | | | |
| Yes (212) | 77 (14.7) | | 135 (13.0) | 1.19 (0.84-1.67) | 0.33 |
| Skin Prick Test response to any of 7 allergens at enrolment [m=35] | | | | | |
| Positive (>3mm) (657) | 241 (42.9) | | 416 (38.0) | 1.26 (0.99-1.61) | 0.06 |
| Allergen-specific IgE at enrolment (N=397)^§^ | | | | | |
| Atopic (>0.35 kU_A_/L) (251) | 94 (67.6) | | 157 (60.8) | 1.17 (0.70-1.95) | 0.55 |
| Total IgE at enrolment (N=398) | | | | | |
| Median (95%CI) kU/L | 421.3 (234.2-596.0) | | 323.3 (271.0-384.8) | 1.00 (0.9998-1.0002) | 0.70 |

N=number; Adj. OR=adjusted odds ratio; CI=confidence interval; SD=standard deviation; m=missing; Columns 2 and 3 represent number (%), unless stated otherwise. ^‡^Adjusted for child’s age, sex, area of residence at birth and father’s education. Test for trend p-value: ^a^=0.12; ^b^=0.48; ^c^=0.002; ^d^=0.08. ^§^ ImmunoCAP^®^, cut-off >0.35 kU_A_/L.
